# Haplotype‐based insights into seminal root angle in barley

**DOI:** 10.1002/tpg2.70088

**Published:** 2025-08-07

**Authors:** Zachary Aldiss, Yasmine Lam, Silvina Baraibar, Sarah Van Der Meer, Eric Dinglasan, Karen Massel, Peter Crisp, Ian Godwin, Andrew Borrell, David Moody, Lee Hickey, Hannah Robinson

**Affiliations:** ^1^ Queensland Alliance for Agriculture and Food Innovation The University of Queensland Brisbane Queensland Australia; ^2^ InterGrain Pty Ltd. Perth Western Australia Australia; ^3^ School of Agriculture and Food Sustainability The University of Queensland Brisbane Queensland Australia; ^4^ Queensland Alliance for Agriculture and Food Innovation The University of Queensland Warwick Queensland Australia; ^5^ Department for Plant Breeding Hochschule Geisenheim University Geisenheim Germany

## Abstract

Root system architecture (RSA) plays a crucial role in crop adaptation and yield stability, especially in the context of climate change and variable growing conditions. Despite this, the genetic basis of RSA remains poorly understood in barley (*Hordeum vulgare* L.), necessitating the need for more research to better characterize this architecture and explore the potential of diverse germplasm for trait improvement. In this study, we aimed to dissect the genetic basis of seminal root angle (SRA) by examining natural variation within a diverse global collection of 816 barley accessions, including both landraces and modern cultivars. Using a haplotype‐based mapping approach, which reflects the recombination patterns considered in breeding programs, we identified chromosomal regions associated with SRA variation. Notably, two major genomic regions on chromosome 5H were identified as novel, while a previously reported region, RAQ1, was confirmed on chromosome 3H. Our analysis revealed significant genetic diversity for SRA within the global collection, with accessions from distinct geographic origins exhibiting unique haplotype combinations. This finding underscores the quantitative nature of the SRA trait and suggests the likelihood of inadvertent selection through polygenic traits related to canopy or yield in commercial breeding programs. To further explore the genetic potential of SRA, we employed a simulation approach to evaluate the feasibility of creating an “ultimate genotype” for narrow SRA. Our results highlight the challenges associated with significantly altering SRA through traditional breeding approaches due to its quantitative, polygenic nature. Consequently, we recommend the integration of predictive and precision breeding techniques, such as genomic selection and gene editing, to effectively capture genetic diversity and accelerate RSA improvement in barley.

## Core Ideas

- First haplotype‐based mapping study for seminal root angle in a diverse global collection of barley.
- Two novel chromosomal regions on chromosome 5H were identified.
- The previously reported region, RAQ1, was confirmed on chromosome 3H.
- Substantial haplotype diversity already present within elite breeding populations.
- Seminal root angle is highly quantitative; combining predictive and precision breeding techniques are likely key to barley improvement.

**Abbreviations**
AGG: Australian Grains GeneBank
BLUEs: best linear unbiased estimates
BLUPs: best linear unbiased predictors
CRISPR: clustered regularly interspaced short palindromic repeats
DH: doubled haploid
DRO1: DEEPER ROOTING1
IGG: InterGrain Core Collection
LD: linkage disequilibrium
LMM: linear mixed model
local GEBV: local genomic estimated breeding value
NRB: Northern Region Barley
PC: principal component
PCA: principal component analysis
QTL: quantitative trait locus
REML: restricted maximum likelihood
RSA: root system architecture
SNP: single nucleotide polymorphism

## 1 INTRODUCTION

Barley (*Hordeum vulgare* L.) is the fourth most important cereal crop cultivated globally (FAS, 2023; Gürel et al., 2016), and well regarded for its adaptability to high‐stress growing environments, an attribute of barley's high level of diversity and genomic plasticity (Cronin et al., 2007; Vanhala et al., 2004). With the increasing globalization of developing countries, there is a higher demand for barley grain to be used for the malting and brewing industries. Additionally, rising meat consumption in these regions drives up the demand for barley as a source for animal feed (van Dijk et al., 2021).

A substantial body of research suggests that optimized root systems can improve water acquisition with minimal metabolic cost, which could improve crop yields while maintaining grain quality under both favorable and adverse growing conditions (Lynch, 2019). The spatial arrangement of roots, known as root system architecture (RSA), is influenced by many growth mechanisms, including root elongation, branching, and curving (Rich & Watt, 2013). These factors collectively determine the distribution of roots through the soil over time, influencing the plant's anchorage and access to water and nutrients (Koevoets et al., 2016). This was illustrated in wheat (*Triticum aestivum* L.) where drought‐adapted lines had a more compact root system, possessing a higher root surface area per soil volume uniformly and allocated more root growth to the deepest soil layers, leading to improvements in yield (Manschadi et al., 2006). Similarly, in rice, the introduction of allelic variation in the *DEEPER ROOTING1* (*DRO1)* gene reduced root growth angle and led to a narrow and deep root system that increased yield under water‐limited conditions (Kitomi et al., 2020). The latter examples in other cereal crops demonstrate the potential for selection of an optimal RSA in barley to improve adaptation and increase production in the face of increased climate variability.

Despite the potential for improved adaptation and yield stability, insights into RSA have been limited due to the phenotyping bottleneck. In‐field, belowground measurements are extremely labor‐intensive, expensive and are typically destructive in nature, and as such are a challenge for large scale phenotyping in breeding programs. As an alternative, glasshouse phenotyping strategies like rhizotrons have been used for RSA observation, but are often limited by glasshouse size and experimental design (Hendrick & Pregitzer, 1996). The clear pot method provides a high‐throughput glasshouse‐based alternative approach to phenotype small‐grained cereal crops for seminal root angle (SRA), which provides a reliable proxy trait for mature root system (Kang et al., 2024; Ober et al., 2021; Richard et al., 2015). Proxy traits such as SRA can be used in conjunction with genome‐wide mapping approaches for quantification and exploration of the underlying genetics of RSA.

To date, a number of studies have explored genomic regions influencing RSA in barley across adapted and diverse germplasm. Most studies have revealed substantial phenotypic variation for seminal root traits. For example, in a population comprising doubled haploid (DH) lines from the ND24260 × Flagship population and Australian cultivars, SRA ranged from 13.5° to 82.2° (Robinson et al., 2016). Quantitative trait locus (QTL) mapping on the DH population identified seven genomic regions associated with seminal root traits across chromosomes 1H, 3H, 4H, 5H, and 6H (Robinson et al., 2016). A major QTL influencing both seminal root angle and number on chromosome 5H (i.e., *qRA‐5/RAQ2*) was also detected in a genome‐wide association study on an Australian barley breeding population (Robinson et al., 2018). Notably, this genomic region is co‐located with *qSDND*, a major QTL for grain dormancy (Comadran et al., 2009; Hickey et al., 2012). Beyond adapted germplasm, Arifuzzaman et al. (2014) detected 33 QTLs for root and shoot traits within a DH population derived from a cross between elite cultivar Scarlett and wild accession ISR42‐8, where almost half of the favorable alleles were donated by the wild parent. A more recent study identified 55 QTLs for RSA in a panel of 221 spring barley accessions, including 55 landraces, phenotyped under controlled conditions (Jia et al., 2019). In a panel constituting only landraces, Khodaeiaminjan et al. (2023) identified five root‐specific seedling QTLs under osmotic stress conditions. Despite the high likelihood of overlap, the genomic regions detected across literature have yet to be directly compared. Furthermore, the value of introducing diverse allelic variation into adapted germplasm is yet to be explored.

In the previous QTL mapping and genome‐wide association studies, a single marker association approach has been the norm and typically a result of lower density genotyping platforms. While useful for broad genomic region discovery, this approach does not consider the relationship between neighboring alleles through linkage disequilibrium (LD) and thus has limitations when used in marker‐assisted selection strategy. An alternative haplotype‐based mapping approach counteracts these limitations, where markers are grouped together based on LD and their cumulative effect on the phenotype calculated, and thus representative of the future chromosomal block inheritance in breeding. The use of the local genomic estimated breeding values (local GEBVs) approach serves as an example of this haplotype‐based approach (Voss‐Fels et al., 2019). To our knowledge, haplotype‐based mapping studies are lacking for RSA in barley.

In the present study, a collection of 816 barley accessions from landraces and Australian modern breeding lines was analyzed to dissect the genetic bases of SRA. The local GEBV haplotype‐based mapping approach was used to identify genomic regions of interest, with a focus on the comparison between global diversity and elite breeding material. Haplotypes with negative, angle‐reducing effects were “stacked” to represent the effects of intensive selection or introgression for the narrow SRA trait in a breeding program.

**Core Ideas**

- First haplotype‐based mapping study for seminal root angle in a diverse global collection of barley.
- Two novel chromosomal regions on chromosome 5H were identified.
- The previously reported region, RAQ1, was confirmed on chromosome 3H.
- Substantial haplotype diversity already present within elite breeding populations.
- Seminal root angle is highly quantitative; combining predictive and precision breeding techniques are likely key to barley improvement.

## 2 MATERIALS AND METHODS

### 2.1 Plant materials

A panel of 816 barley accessions, consisting of a mixture of spring and winter, and two‐ and six‐rowed lines, was characterized for SRA. Within the panel, 384 of the lines were a subset of the Australian Grains GeneBank (AGG) and the remaining 432 were a subset of the InterGrain Core Collection (IGG). The AGG collection represents the global diversity of barley, including wild and modern cultivars alongside landraces, a derivative of the international barley core collection first established in 1992 and brought to Australia in 2000 (van Hintum, 1994). Conversely, the IGG is a representative collection of the genetic diversity of Australian barley breeding across the last decade, consisting of two‐row spring breeding lines and commercial cultivars. All lines in the panel were previously genotyped with 12,562 unique markers using the Illumina Infinium 40K XT SNP bead chip (Keeble‐Gagnère et al., 2021). Genotypes were encoded in numeric variant call format as diploid genotypes, with the reference allele followed by the most common alternate (A = 1, C = 2, G = 3, T = 4) (Danecek et al., 2011). Missing marker values for each genotype of the AGG panel were imputed using a low‐rank approximation using nuclear‐norm regularization through the R studio package softImpute (Mazumder et al., 2010). Imputation for the IGG panel was performed by the genotyping service provider using Beagle5.4 (Browning et al., 2018). Alternating approaches were used due to the diversity in the AGG panel and the potential for bias using an unrelated reference population in the imputation.

### 2.2 Root angle phenotyping

The “clear pot” method (Richard et al., 2015) adapted to barley (Robinson et al., 2016) was used to evaluate SRA, where SRA is defined as the average deviation angle of the first pair of seminal roots to the vertical plane (Christopher et al., 2013; Richard et al., 2015; Robinson et al., 2016).

Due to the size of the panel and number of replicates, the experiment was subset into three sub‐experiments, where appropriate concurrence was maintained for downstream multi‐experiment statistical analysis. Each experiment had six replicates for each genotype across 96 pots, containing 24 seeds per pot using the panel's relatedness, as determined by the genomic relationship matrix, as weighting of samples in a resolvable incomplete block design. Each block consisted of 32 pots spread out in a 2D array of 12 columns × 8 rows, blocked in the column direction with two columns per replicate. Experiment design was generated under a linear mixed model (LMM) framework where lines were biased against genetic similarity using a genotype relationship matrix using the R packages Optimal Design and Pedicure, an extension of ASReml‐R version 4 (Butler et al., 2017).

Seeds were sown in 4 L ANOVApots of 200 mm diameter and 190 mm in height. Seeds were sown at a depth of 2 cm at a 45° angle, with the embryonic axis of each seed oriented downward, every 2.5 cm along the transparent wall of the pot to result in 24 barley seeds per pot (Robinson et al., 2016). UQ‐23 potting mix was used, containing 70% composted pine bark (0–5 mm) and 30% cocoa peat. Plants were housed in a temperature‐controlled glasshouse, with 22°C/17°C (day/night) temperature cycle and an 18 h/6 h (day/night) photoperiod with natural lighting and growth lights to supplement.

The pots were thoroughly watered the day before sowing and no additional water was provided, which ensured the orientation of seeds did not shift during germination. After sowing, each clear pot was placed inside a black pot of the same dimension to prevent light from influencing root development. Images of roots were captured at 5 days after sowing using a camera when roots reached approximately 5 cm in length (iPhone 13 Pro Max). Analysis for SRA was determined using ImageJ (Schneider et al., 2012).

### 2.3 Statistical analysis

A multi‐experiment analysis was performed where an LMM was fitted to account for spatial variation and maximize the genetic variance captured in each trial. The LMM was fitted using the following equation:

y=Xτ+Zu+ε

where *X* and *Z* are design matrices associated with fixed (*τ*) and random (*𝑢*) effects and *𝜀* is the residual errors. The fixed and random effects deemed significant in single experiment analysis were fit in the multi‐experiment model to account for spatial variability. The genotype by experiment interaction term was then explored for the best variance structure fit using a restricted maximum likelihood (REML) approach. The goodness of fit was determined by the Wald test statistics (for fixed effect terms only), the highest REML log‐likelihood value, and the smallest Akaike information criterion and Bayesian information criterion (Akaike, 1974; Kenward & Roger, 1997) (Supplementary Table 1).

For SRA, an unstructured variance structure was used, and linear column and row effects were fitted as fixed effects, while column, row, and pot were included as random effects (Table S1). The auto‐regressive residual variance structure in the order of one was fitted to the residuals. The LMM was fit using ASReml‐R in the R software environment (R Core Team, 2024). The best linear unbiased estimates (BLUEs) and best linear unbiased predictors (BLUPs) were calculated using genotype as a fixed and random effects, respectively. A generalized measure of broad sense heritability was calculated to quantify the proportion of phenotypic variance that is attributed to genetic effects. The Cullis method was used to account for the unbalanced population distribution across the three trials (Cullis et al., 2006). This was determined using the following equation:

$$H_{\mathrm{Cullis}}^{2}=1-\frac{{\bar{v}}_{\Delta }^{\mathrm{BLUP}}}{2\times \sigma_{g}^{2}}$$

where $\sigma_{g}^{2}$ is the genotypic variance and ${\bar{v}}_{\Delta }^{\mathrm{BLUP}}$ is the mean standard error of the genotype BLUPs. Figures were created using the ggplot package in R (Wickham et al., 2016). As seminal root angle follows a continuous distribution, a variety was classified as having a narrow angle if it was less than the mean minus 1.5 times the standard deviation and wide angle if it was greater than the mean plus 1.5 times the standard deviation, respectively.

### 2.4 Population structure

Single nucleotide polymorphism (SNP) marker curation was performed, removing markers with a missingness and heterozygosity of greater than 10%. Due to the high diversity of the collection, a minor allele frequency of >1% was used to capture rare alleles in the population. As a result, 11,646 high quality polymorphic SNP markers with good chromosome coverage (Figure S1) were identified for downstream analysis.

Principal component analysis (PCA) of the SNP data was performed to explore population structure among the 816 barley accessions. Specifically, multidimensional scaling was performed based on genetic distance matrices calculated using Rogers Distance (Rogers, 1972). The Ward 2 error sum of squares hierarchical clustering method was used to classify lines into clusters of relatedness (Murtagh & Legendre, 2014).

### 2.5 Haplotype‐based mapping of seminal root angle

SRA BLUEs and the SNP marker profiles of the panel were used in a haplotype‐based approach to calculate local GEBVs. A haplotype mapping approach was selected as it provides more trackable and achievable targets for plant breeders than QTL mapping or genome‐wide association studies due to its incorporation of LD and its ability to more successfully capture weak signal QTLs that may be lost through genome‐wide association studies (Brunner et al., 2024; Voss‐Fels et al., 2019). Prior to analysis, genome‐wide SNP markers were assigned to blocks of high LD based on pairwise *r* ^2^ with a minimum LD threshold of 0.3. This threshold was selected due to the lower level of LD within the diverse population, where the genome‐wide rate of decay was 0.028 (Figure S2). Markers within this threshold or above were considered to be in the same block, while markers below are considered new blocks. A marker tolerance of *t* = 3 was set per block to account for incorrectly positioned markers. As such, when at least *t* flanking markers had lower than 0.3 *r* ^2^ LD threshold, the block is complete. This process was used to establish the blocks genome wide, resulting in 3347 haploblocks with an average of 3.5 markers per block.

To identify chromosomal regions with a large impact on SRA, the effect values were assigned to the LD blocks, termed haploblocks. Marker effects within each block were predicted using the ridge regression linear unbiased prediction model and summed to calculate the haplotype effect. (Meuwissen et al., 2001). The variance in haplotype effect for each haploblock was calculated and correlated with the number of SNPs within each haploblock to ensure that there is no bias between the number of SNPs and variance for each haploblock (Figure S3). Haploblock variances were plotted on a Manhattan plot to identify the blocks with the highest variance for block effects. Block variance was log scaled and plotted against the range in haplotype effect for each haploblock. For the top 0.5% of high variance blocks, the start and end physical positions were collected and positioned against known QTLs and genes regulating root traits. A physical map was generated using MapChart (Voorrips, 2002).

### 2.6 Simulated haplotype analysis

For downstream haplotype analysis, the top 0.5%, 10%, and 100% of blocks with the highest scaled variance were selected. To contrast the potential improvement in SRA across both germplasm pools, a simulation approach was employed. Using only the genetic information from the IGG commercial breeding population, the maximum negative effect, favoring a narrow SRA, for each haploblock was selected and stacked to explore its cumulative phenotypic effect. This process was then repeated with AGG and then the combined dataset, both IGG and AGG genetics, to consider the broader genetic diversity offered by the global diversity panel.

## 3 RESULTS

### 3.1 Wide variation for SRA across the AGG and IGG collections

**FIGURE 1:**
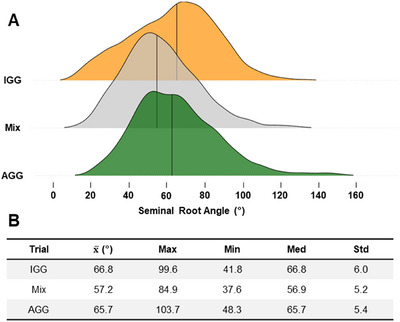
Seminal root angle variation across the panel of barley accessions. (A) Density plot showing a smoothed distribution of the seminal root angle (SRA) trait captured across three trials; predominately InterGrain Core Collection (IGG) (orange), Mix (gray), and predominately Australian Grains GeneBank (AGG) (green). The vertical line indicates mean SRA. (B) Summary table for adjusted results (best linear unbiased predictors [BLUPs]) of each trial following spatial analysis. From left to right, the columns indicate the population included for each experiment, the mean SRA, the maximum SRA, the minimum SRA, the median, and the standard deviation.

In total, 816 accessions were phenotyped for SRA with a broad‐sense heritability of 0.65 from the multi‐experiment analysis. The BLUEs across environments revealed 10% of accessions showed wide‐angled phenotypes (SRA > 75°) with a maximum of 103.7°, 8% showed narrow root phenotypes (SRA < 55°) with a minimum of 39.7°, and 72% displayed a moderate root angle phenotype with an SRA greater than 55° and less than 75° (Figure 1). Of those displaying narrow root angles, 66% were from the AGG collection, while for those with wide root angles, AGG represented only 22% of accessions. The accession with the most extreme narrow phenotype was from the IGG collection, while the accession with the widest root angle was from the AGG collection. The greatest variance came from the IGG population with a trial mean of 66.8° and variance of 36° compared to AGG with a trial average of 65.7° and a variance of 29°.

### 3.2 Distinct population clusters within IGG and across the entire collection

**FIGURE 2:**
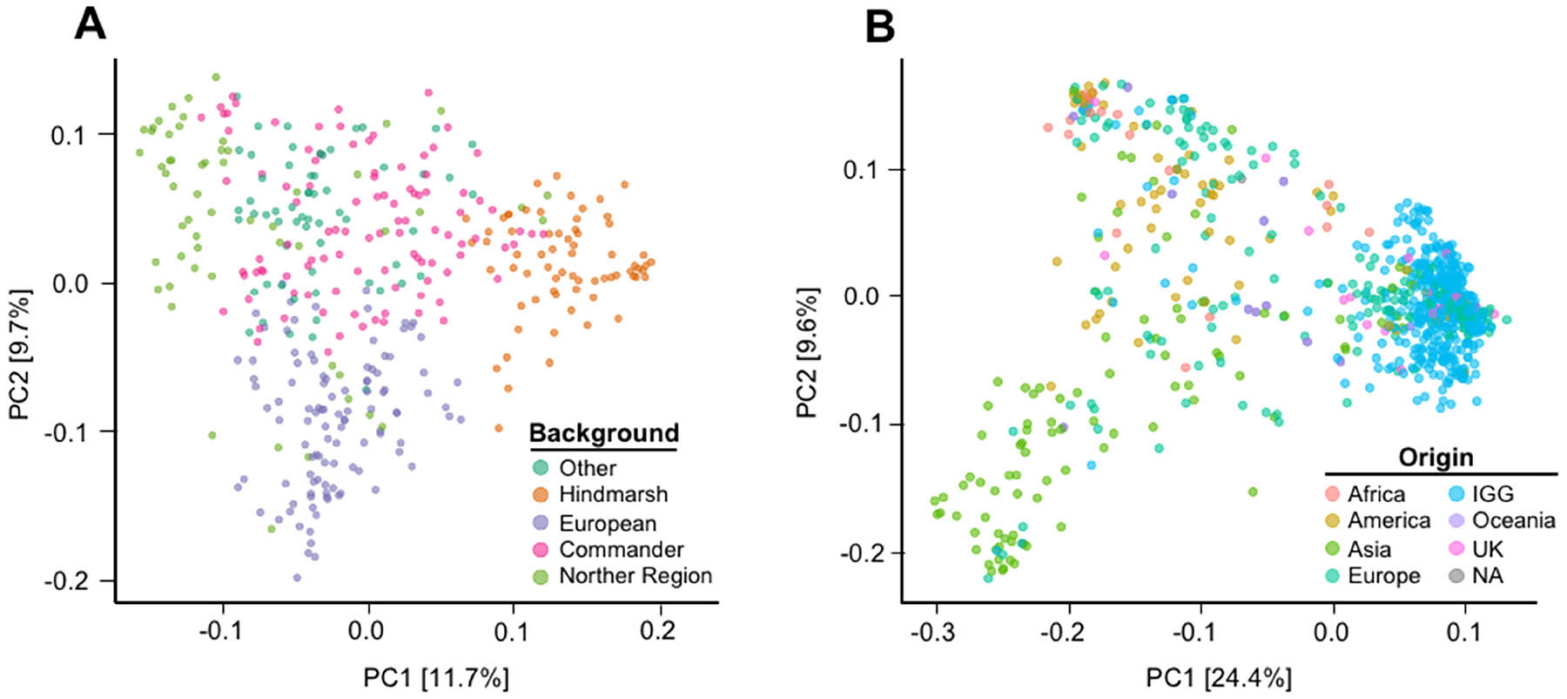
Visualization of population structure (principal component analysis [PCA]) of the InterGrain Core Collection (IGG) and Australian Grains GeneBank (AGG) + IGG collections. (A) Visualizing the genotypic diversity of the IGG collection. Clustering into those of European origin, Australian Northern Region Breeding, Commander, and Hindmarsh varieties. PC1 and PC2 (where PC is principal component) explains 11.7% and 9.7%, respectively. (B) Genetic relationship of AGG and IGG accessions where PC1 (24.4% of variation) and PC2 (9.6% of variation). PCA of AGG and IGG collection visualizes the continent of origin for each accession.

A PCA was performed to explore population structure within and across groups. The first principal component (PC) captures 11.7% of the variance for the IGG collection (Figure 2A) compared to 24.4% of the IGG + AGG collection (Figure 2B). Thus, more population structure is found in the combined population as more variation is explained by the first PC.

The PCA highlights the distinct populations present within the collections, coinciding with the accessions geographic origin. Hierarchical clustering analysis of IGG revealed four clusters aligning with four subgroups: those of European origin, Australian Northern Region Breeding, Commander, and Hindmarsh origins. A similar clustering analysis on the combined population formed three major subgroups, with the accessions from Asia appearing the most distinct (Figure S4). Meanwhile, the largest spread of variance was with the European varieties, clustering with the IGG collection. Interestingly, the Americas have a uniquely diverse collection of germplasm being present alongside and distant from European lines. This aligns with the distribution of row type into two major groupings.

### 3.3 Chromosome 5H contains the highest variance blocks for SRA

The highest variance block was detected on chromosome 5H (b001908), with 258 SNPs within the haploblock. No correlation between the number of SNPs and the variance explained was detected (Figure S3). At a threshold of 0.5%, the top 16 highest variance blocks were identified as the primary focus for further analysis. The second highest variance block was also found on 5H (b001923). b001908 exhibited the widest range of haplotype effects on GEBVs, with estimates spanning from −0.6° to 0.6°. The second largest block, b001923, also showed both negative and positive haplotype effects on GEBVs, skewing more toward larger positive effects with a range of −0.3° to 0.4°. Overall, a good range in variance for haploblock effects was detected in this analysis showing both positive and negative haplotypes.

### 3.4 High variance haploblock co‐located with known root angle QTL *RAQ1*

Across the diversity represented in both collections, the top 0.5% of blocks were aligned with previously identified QTLs in the literature (Figure 4; Table S2). The highest variance blocks, b001908 and b001923 on 5H, were novel, positioned toward the center of the chromosome. Of the significant blocks, b001027, located on 3H and comprised of 115 SNP markers co‐located with known root angle QTL *RAQ1*. The largest effect block, b001908, aligned with the known root angle gene *EGT2* and contained 258 markers, spanning approximately 194.44 Mbp, with an overall variance of 0.14. While there were no markers directly positioned on EGT2 with the closest flanking markers being 1 Mbp from the gene, these are highly linked due to being within the linkage block (Figure S5). All other significant blocks did not align with known QTLs and were considered novel. Interestingly, a number of the top blocks were identified in the centromeric region of the chromosomes (e.g., b000083, b000527), where previous studies have predominantly identified associations on the proximal and distal ends of the chromosomes. Additionally, candidate genes associated with root traits and development did not co‐locate with any major haploblocks (Figure 4) a full list of haploblocks and candidate genes can be found in Table S3. Interestingly, b001538 on 4H collocated with vascular patterning gene *PIN1b* (HORVU4Hr1G026680), although effects on root architecture in barley are currently unknown.

### 3.5 Block variance clustering reveals shared and population‐specific variation

Contrasting the block variances of the IGG and AGG populations revealed the highest variant block, b001908, shared its high haploblock variance in both populations. Similarly, b001923, the second highest block overall, follows a similar trend with only slightly higher variance in the IGG collection (Figure 5A). To explore the variance of the smaller effect blocks, the variance was log‐scaled. Smaller effect blocks separated out into two subgroups, those with high variance in both the diverse AGG collection and the IGG breeding population, in the top cluster, and those exclusive to the AGG collection clustering at the bottom (Figure 5B). As indicated by color, the top cluster has a higher proportion of negative haplotype effect blocks (yellow), while the bottom cluster has a more even spread of positive (blue) and negative effects.

### 3.6 Haplotype effects cluster based on population structure

The origins of the haplotypes for the highest variance block b00908 and the haploblock collocating with known root angle QTL *RAQ1* were explored through the population structure described by the PCA. For b001908, in the IGG population, the strongest negative haplotypes originate from the Hindmarsh background, while the most positive effects originate from the northern region cluster (Figure 6A). Haplogroups were also created based on flanking markers of EGT2 located within b001908.There were two major haplogroups, CC‐CC and CC‐TT, capturing 47% and 44.4% of the population, respectively (Figure S7A). Haplogroup CC‐TT was significantly associated with GEBVs for SRA, showing an approximate 2° increase compared to the reference group CC‐CC (Figure S7B). However, there was no significant association between haplogroup frequency and population. In the AGG global diversity, the strongest negative effect haplotypes originate from Europe with the remaining positive effect haplotypes appearing in European and Asian accessions (Figure 6B). *RAQ1* showed widespread presence of positive effect haplotypes in the IGG lines, while the strong negative effect haplotypes were rare but tended to appear more frequently in the European accessions (Figure 6C). In the AGG collection, *RAQ1* shows null effect haplotypes in both Asian and some European subgroups, while the strong negative effect haplotypes cluster in the Americas (Figure 6D).

### 3.7 Targeted haplotype stacking for “ultimate” narrow rooting genotype

Of the 3347 haploblocks detected, 2815 had haplotype effects conferring a narrow SRA and reducing the mean estimated GEBV for SRA. Selection of the 0.5% of the highest variance blocks produced a mean shift in the GEBV for SRA of approximately −3° for the stacking of 16 blocks, with no difference in GEBV across the three genetic pools (Figure 7A). This outcome was similar following selection of the top 10% of blocks, producing a shift of −15° for all populations, with a slight difference in the GEBV for SRA beginning to appear between AGG and IGG at 280 blocks (Figure 7B). The ultimate narrow root angle genotype was simulated by incorporating all available highest effect haplotypes for both populations, shifting the mean GEBV for SRA by approximately 30° (Figure 7C). Using IGG genetics alone shows a maximum shift of 27° (Figure 7C). There was no difference between IGG + AGG and AGG alone, suggesting the additional 4° is coming from exclusively AGG genetics. The SRA haploblocks are highly quantitative with the difference in diversity between AGG and IGG beginning to show after stacking approximately 100 blocks, with the maximum difference in GEBV for SRA present after approximately 2000 blocks before reaching a plateau.

## 4 DISCUSSION

In a comparison of the two groups, the diverse AGG collection displayed a higher frequency of wide SRAs, while the IGG breeding collection was skewed toward narrower SRAs. This is not surprising, based on the broad global genetic diversity that the AGG collection captures compared to the IGG that clusters with the European germplasm within the AGG (Figure 2B). This is also a potential reflection of inadvertent selection and adaptation toward the dominant growing environments within Australia, where a narrow and deep RSA may be beneficial. However, it is important to note that although skewed toward a narrower SRA, wide variation was still observed for the IGG collection, where SRA ranged from 41.8° to 99.6° (Figure 1B).

Even narrower SRA phenotypes were observed in the previous studies examining Australian germplasm using the same phenotyping method. For example, in the ND24260 × Flagship DH population, SRA ranged from 16.4° to 70.5° (Robinson et al., 2016). Similarly, SRA ranged from 12.0° to 89.4° in elite germplasm from the Northern Region Barley (NRB) breeding program (Robinson et al., 2018). While the range of approximately 70° is consistent for both the breeding populations, the maximum and minimum are shifted by approximately 20°. Again, this could reflect inadvertent environmental adaptation whereby the IGG program typically breeds for broad adaptation across Australia, while the NRB program specifically targets the northern grain‐growing region. This region has a unique growing environment, characterized by high clay content soils with high water‐holding capacity, and high frequency of summer rains yet minimal growing‐season rainfall. As a result, crops are typically reliant on stored soil moisture throughout the growing season. This suggests that the NRB population may have narrower RSA to access deep stored soil moisture, similar to the previously described “Steep, Cheap, and Deep” ideotype (Lynch, 2013). This also suggests the allelic variation driving this narrow RSA in the Australian NRB and DH population may not be captured in the present study and is likely originating from the North Dakota breeding germplasm ancestry common in both populations. Future research combining a selection of the NRB breeding lines with the panel examined in the current study could be beneficial to explore genomic regions influencing narrow SRA from a haplotype‐based mapping approach.

A comparison of the phenotypic diversity of the AGG collection, compared with a diverse spring barley collection composed of cultivars, landraces, and breeding lines from 51 countries, revealed similar phenotypic variation in SRA. Whereby, the AGG collection SRA ranges from 48.3° to 103.7° with a mean of 65.7°, and the spring barley panel ranges from 46.7° to 131.7° with a mean of 70.5° (Jia et al., 2019). While the minimum and mean of the SRA for both populations are similar, the maximum observed is higher in the spring barley diversity panel. This difference may be due to variations in the phenotyping methodology, particularly the later date and greater depth at which measurements were taken. However, this may also reflect the fact that most accessions in the spring barley panel are from Europe, which has cooler temperatures and higher in‐season rainfall compared to the African and Australian varieties in the AGG collection. In comparison, commercial lines bred for Nordic climates were recently examined for SRA and found to have a mean of 95°, with a range from 43.2° to 151.3° (Smith et al., 2024). It is important to note that while these trends align with previously reported ideotypes of wide RSA being beneficial for shallow light soils with high in‐season rainfall and narrow RSA in converse environmental conditions (Lynch, 2013), the benefits of these ideotypes within these environment types are yet to be confirmed. Quantifying the value of different RSA in specific growing conditions for improved yield stability is critical for breeding. However, this remains underrepresented in the literature due to the challenges associated with reliably measuring below‐ground RSA.

Beyond commercial cultivars, landraces and domesticated relatives have been evaluated for centuries in local agricultural communities where they have been adapted to specific environmental conditions. This global pool of diversity, represented by the AGG collection, provides a significantly larger variation in genotypes for potential introgression into elite breeding material, represented by the IGG collection. The population structure supports this, with the majority of IGG material grouping with the AGG lines of European origin. This is consistent with the origins of barley cultivation in Australia, where at the turn of the 19th to 20th century, two‐rowed spring landrace selections from the UK were introduced (Seko, 1987). This approach has continued, with donor material brought into Australia from Europe, such as cultivars Archer or Chevallier, as the germplasm is adapted to higher rainfall zones (Baumer & Cais, 2000).

**FIGURE 3:**
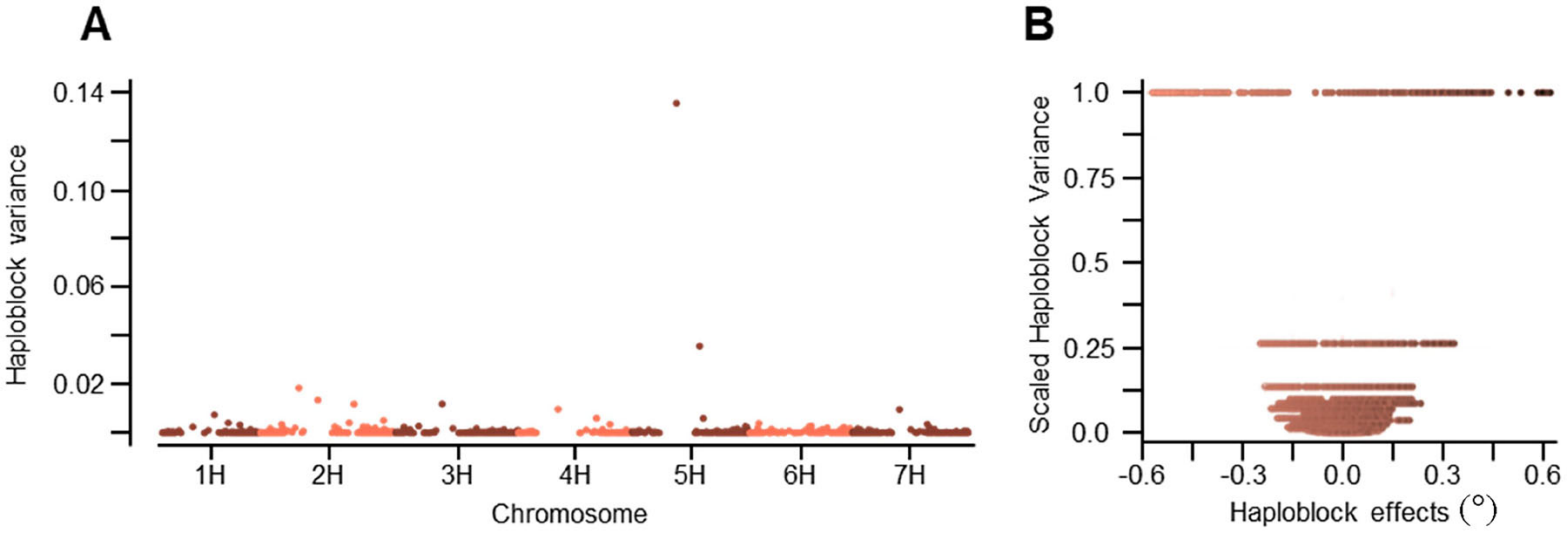
Haploblocks with high variance for effect on GEBVs for seminal root angle. (A) Manhattan plot displaying the high variance for haploblock effects. Each point represents a haploblock, where the *x*‐axis is the genomic position on each chromosome and the *y*‐axis indicates haploblock variance where higher variance indicates a greater range of haplotype effects, thus greater genetic influence on the trait. (B) Scatter plot illustrating the relationship between haplotype effects and scaled block variance for each haploblock. The *x*‐axis represents the haplotype effect where each dot is an individual haplotype, while the *y*‐axis represents the scaled variance of each haploblock.

**FIGURE 4:**
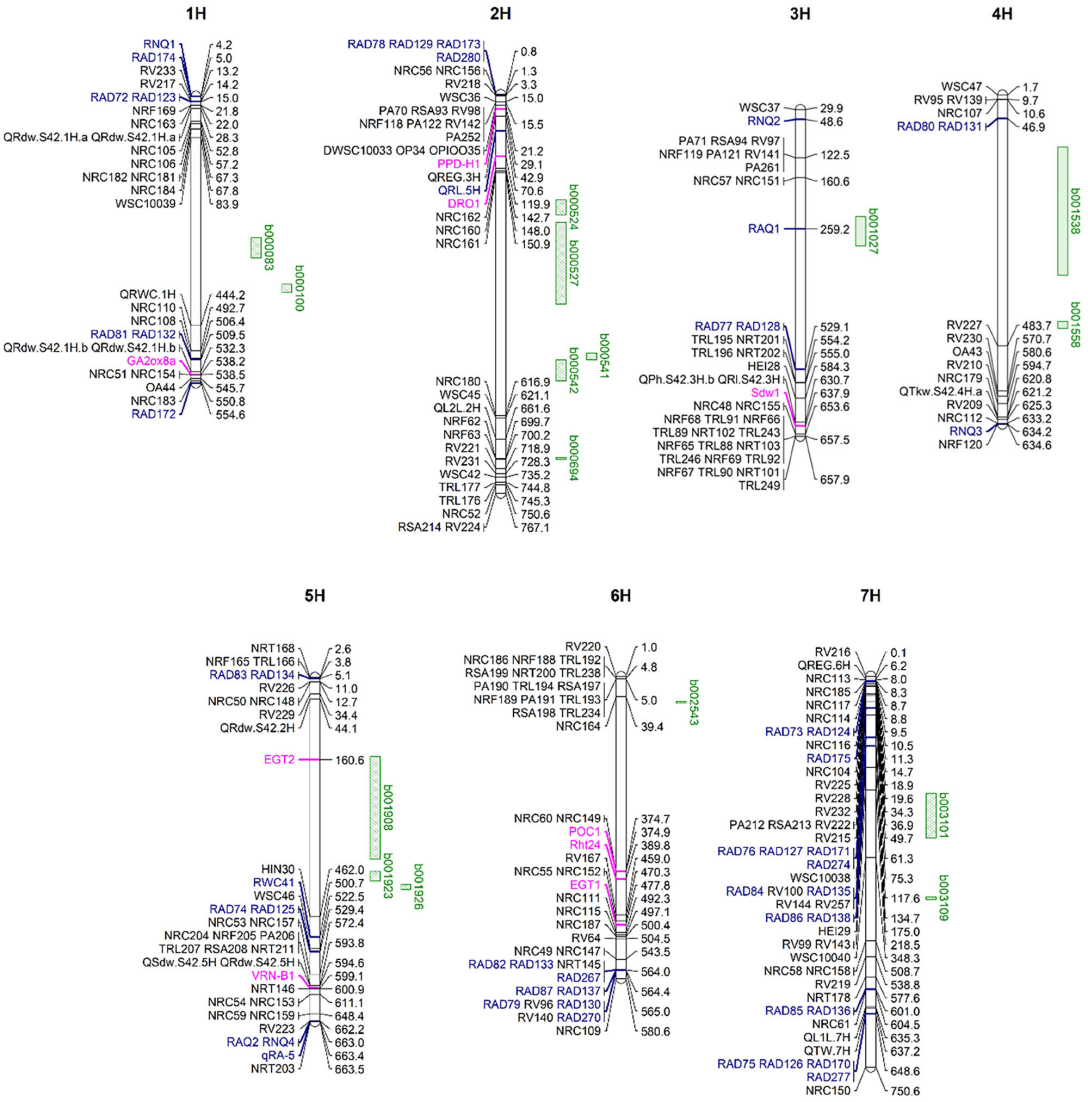
Alignment of discovered haploblocks with previously reported root trait quantitative trait loci (QTLs). Previously reported QTLs are listed on the left side of the chromosome with the corresponding physical position (Mbp) shown on the right. Highlighted in green are the significant haploblocks. Those that do not align with known root trait QTLs are considered novel. b001027 aligns with *RAQ1* and b001908 aligns with *EGT2*. All blue markers on the physical map are specifically associated with root angle or number. Pink highlights known genes regulating root or aboveground development. Full marker information is listed in Table S3 (Arifuzzaman et al., 2014; Chen et al., 2009; Cheng et al., 2023; Chloupek et al., 2006; Diab et al., 2004; Siahsar & Narouei, 2010; Fusi et al., 2022; Kirschner et al., 2021; Robinson et al., 2016, 2018; Siddiqui et al. 2024; Teulat et al., 2001; Uga et al., 2011; Voss‐Fels et al., 2018).

Within the top 0.5% of the highest variance haploblocks, b001027 on 3H was found to collocate exactly with the previously reported QTL, *RAQ1* (Figure 4). Within the IGG population, one of the highest effect haplotypes for *RAQ1* is present within Flagship, the malting variety where *RAQ1* was originally detected (Robinson et al., 2016). Additionally, many of the larger top blocks were in the centromeric region of the chromosomes (Figure 4), a result of the haplotype mapping incorporating LD, where recombination occurs at lower rates in the more central regions of the chromosomes, resulting in larger blocks. When exploring the effects across the entire IGG collection, the strongest negative effect trends toward the cluster with European origins, while sporadic in its distribution, the remaining accessions in the panel have strong positive effect haplotypes, demonstrating the negative effects are rare (Figure 6B). AGG has a higher proportion of null or negative effect blocks, with the positive effects clustering toward African and American origins while the negative and null effects are clustering in Europe, consistent with IGG and Asia (Figures 3B and 6B). Interestingly, while *RAQ1* explained 3.8% of phenotypic variation in the double haploid population, b001027 aligning with RAQ1 explains a significantly lower percentage, only 1.1%, likely reflecting the higher genetic diversity within the AGG and IGG collections (Table S2).

While the top two highest variance blocks were found on 5H, the chromosome that contains several previously detected seminal root trait QTLs, they do not align with these known regions and are considered novel (Robinson et al., 2016, 2018). This may be due to the significant differences in diversity of the collections, where previous studies examined a DH population and a breeding program targeting the Australian Northern Grain Growing region (Robinson et al., 2016). While DH populations enhance the statistical power to detect rare alleles through an increase in allelic frequency throughout the population, they are limited to the genetic diversity present within the two parents.

**FIGURE 5:**
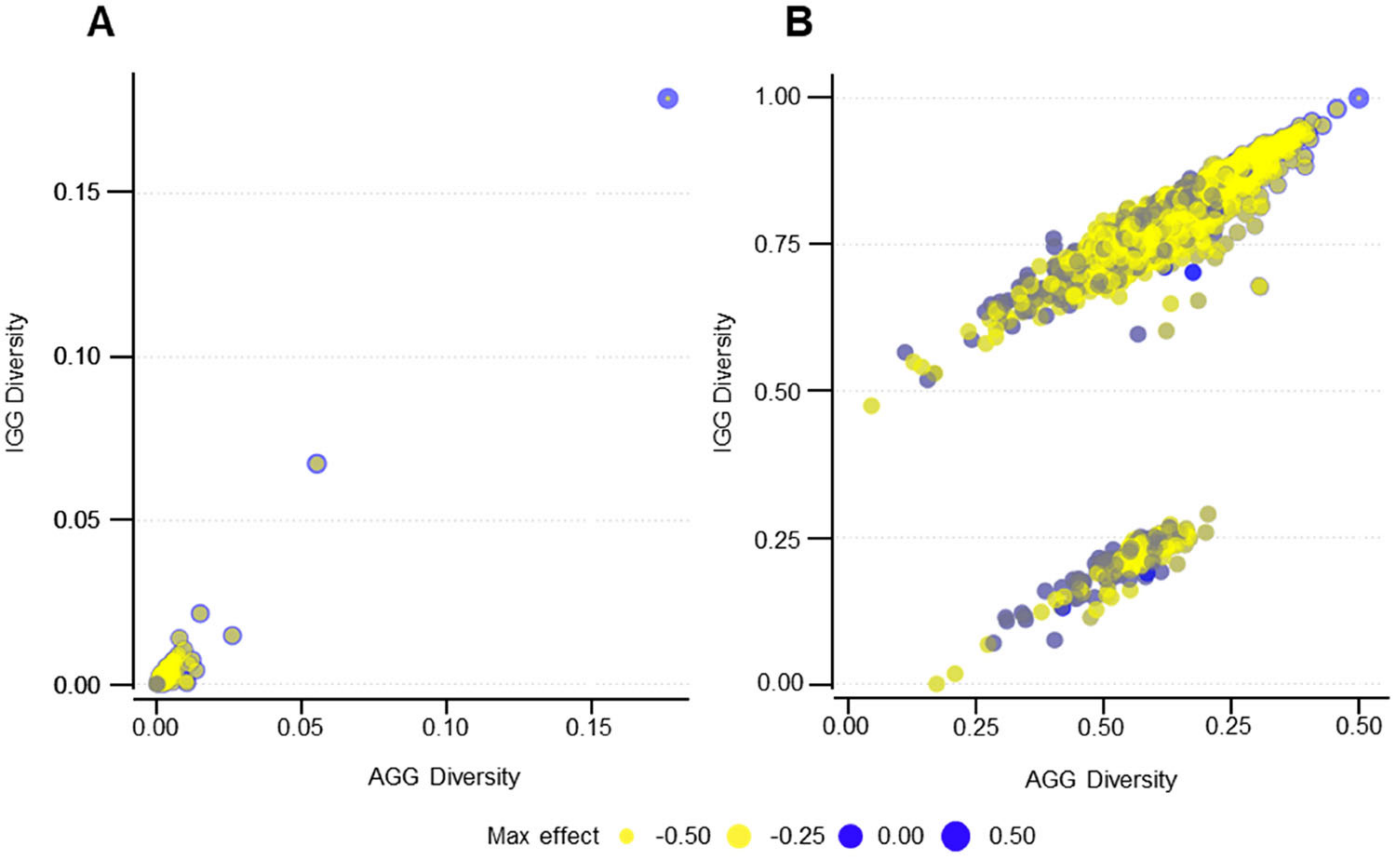
Comparing haploblock variance between the diverse Australian Grains GeneBank (AGG) collection and the InterGrain Core Collection (IGG) breeding population. (A) The raw variance of each haploblock for both the IGG collection (*y*‐axis) and the AGG collection (*x*‐axis). The haploblock effect variance is on a linear scale, and higher values indicate a stronger influence of the haploblock on the GEBVS for seminal root angle (SRA). (B) Log‐scaled variance of the values from A, particularly to explore the lower variance blocks without the skew caused by the highest variance blocks. The point size represents block effect in both panels, with yellow representing negative effects and blue representing positive effects on GEBVs for SRA.

Comparing the haploblock variances between IGG and AGG, the highest variance blocks, b001908 and b001923, have equal variance for marker effects in both populations, demonstrating a strong presence in both the IGG and the AGG (Figure 5A ). This suggests that although the IGG breeding program has not specifically targeted RSA, they may have likely unintentionally selected for RSA through the selection of aboveground polygenic traits crucial for adaptation, such as flowering time and yield. The presence of these high value haploblocks within the IGG collection is highly advantageous for breeding and simplifies selection, since there is no requirement for labor‐intensive introgression. While high variance blocks associated with SRA have equal variance in both collections, smaller effect blocks separate into two distinct clusters (Figure 5B). Interestingly, the bottom cluster consists of haploblocks that have low variance in the IGG collection yet high variance in the AGG panel, demonstrating its contribution of allelic diversity (Figure 5B). The reflected diversity of this cluster is a prime target for introgression into the IGG breeding collection; however, the value of such is unclear and likely minimal given the small effect sizes of the haplotypes.

**FIGURE 6:**
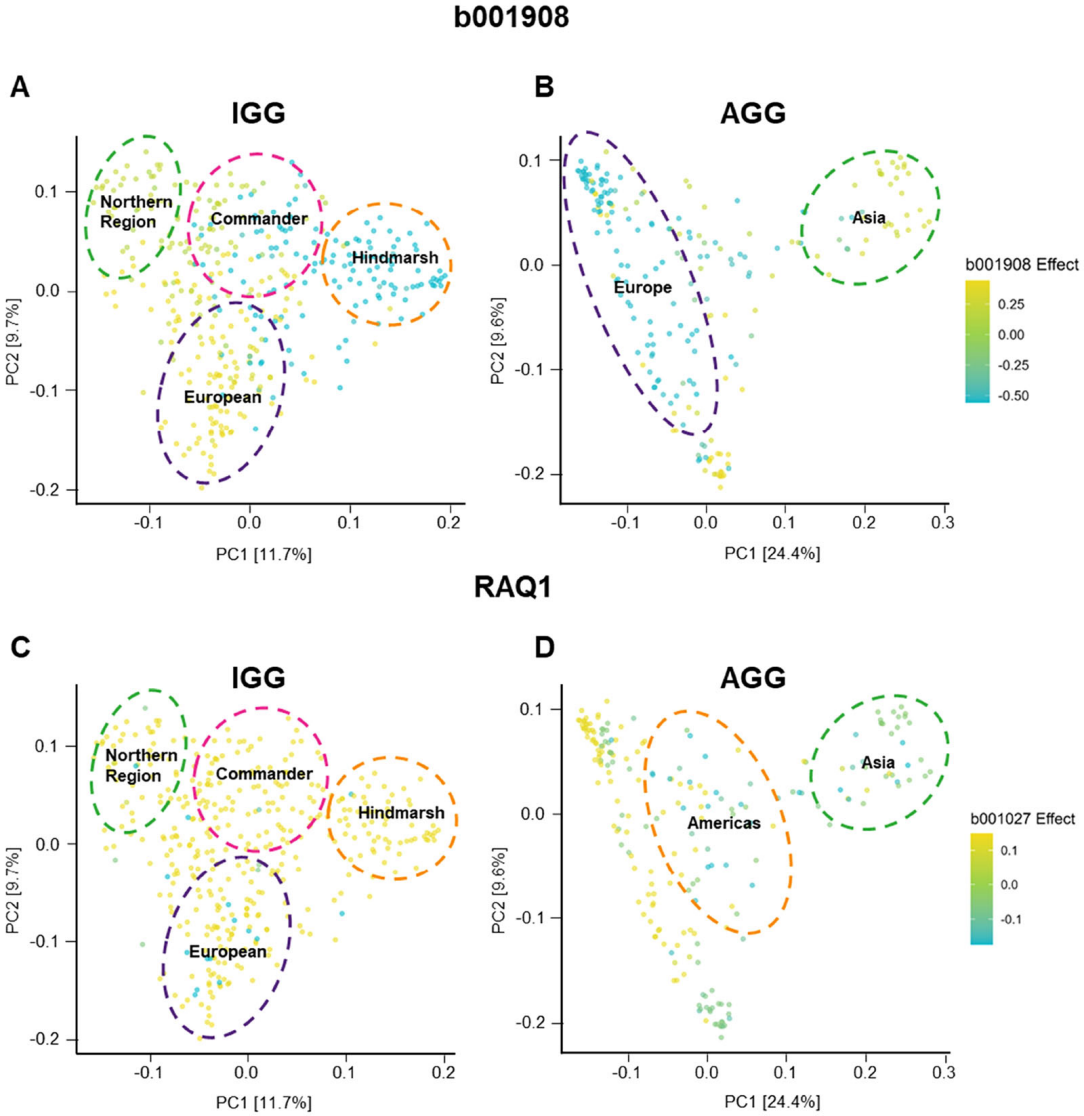
Principal component analysis (PCA) of highest variance and co‐located haploblocks shows haplotype effect with comparisons to accession origins. Each point represents a line from either the InterGrain Core Collection (IGG) panel (left) or the Australian Grains GeneBank (AGG) panel (right) and is colored by the effect size and direction of the line's individual haplotype within the haploblock. Positive effects (yellow), zero effect (green), and negative effect (blue) are plotted for b001908 (A and B), the highest variance haploblock located on 5H for IGG and AGG populations, and b001027 (C and D), which collocated with known root angle quantitative trait locus (QTL) *RAQ1* on 3H, haplotype effects colored on IGG and AGG population structures with origin circled.

**FIGURE 7:**
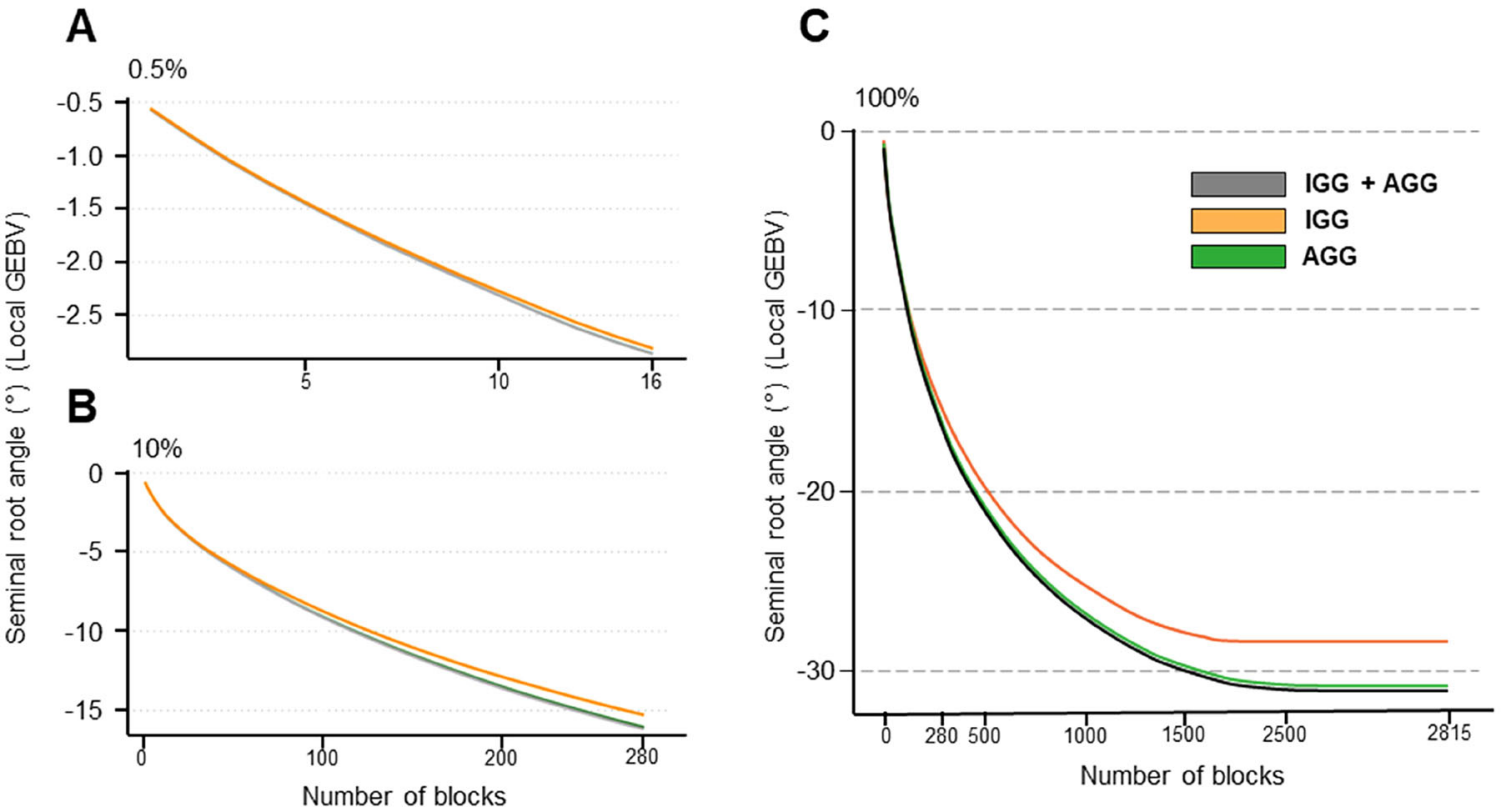
Haploblock stacking analysis for narrow root angle. Haplotype blocks present in three conditions, utilizing only InterGrain Core Collection (IGG) germplasm (orange), only Australian Grains GeneBank (AGG) (green), and all available germplasm (AGG and IGG; gray), for the genomic region associated with seminal root angle (SRA) are selected in order of highest variance. The *x*‐axis represents the number of blocks fixed at the highest haplotype effect for that block. The *y*‐axis is the mean change in predicted SRA (GEBV) through cumulative addition of negative effect haplotypes. (A) Visualizing the mean change in GEBV through selecting the top 0.5% (top) and (B) 10% (bottom) of haploblocks sorted by variance. (C) The ultimate genotype produced through stacking all 2815 haploblocks with conferring narrow SRA GEBV.

Through a simulated approach, we can explore the effects of cumulatively staking the maximum effect haplotypes to develop the “ultimate genotype” (Hayes et al., 2024; Kemper et al., 2012), where here we focus on developing a narrow RSA following the “Steep, Cheap, and Deep” ideotype. Using this approach, we demonstrate the quantitative nature of the trait, as it requires over 100 blocks to detect a difference between AGG and IGG collections (Figure 7B; Figure S5). Considering the complexity of the trait and the genetic architecture of similar traits, the highly quantitative nature of SRA is not surprising, but it does make a traditional marker‐assisted selection approach unfeasible. A whole‐genome approach, such as genomic selection, is more efficient and would allow downstream cross‐prediction, where artificial intelligence is used to optimally select high value parents for breeding of optimal RSA (Villiers et al., 2024). One such approach incorporates haplotype selection into a genetic simulation where parent selection can be specifically determined for breeding the ultimate genotype, in this case for a narrow RSA (Villiers et al., 2024). However, the potential for modifying this trait is limited. Using all available genetics within the IGG collection, the maximum trait shift is −27°. When combining both IGG and AGG, the shift is −30°, demonstrating that even with global diversity, the SRA cannot be significantly altered beyond what is already found in nature.

To make large modifications to the RSA, the mechanistic pathway likely needs to be unveiled and then exploited. In this context, key genes in the root developmental pathway that may have minimal allelic variation in current barley germplasm need to be discovered. If they have a large effect, these genes could be targeted to create beneficial variation using biotechnological tools such as clustered regularly interspaced short palindromic repeats (CRISPR) genome editing. While there is still limited knowledge of the molecular pathways influencing SRA in cereals, knowledge of developmental genes in model species such as Arabidopsis could be leveraged to accelerate progress in barley (Kong et al., 2014; Zhang et al., 2018). Recent examples include knockout of a single gene (*PIN‐FORMED 2*) in barley resulting in reduced gravitropic response and shallower root architecture with an increase in root angle by over 100% (Aldiss et al., 2024). Alternatively, the reduced function of *EGT2* results in an increase in gravitropic response, causing a reduction in root angle by 50% (Kirschner et al., 2021). A similar approach has been illustrated in rice, where introduction of allelic variation in the *DRO1* gene decreased root growth angle and led to deeper rooting plants and increased yield under water‐limited conditions (Kitomi et al., 2020). With advancements in genomic selection and gene editing technologies, these predictive and precision breeding tools create a toolbox that can be exploited to enhance genomic breeding. This integration has the potential to fast‐track cereal crop improvement, enabling rapid adaptation to changing growing conditions (Feng et al., 2017).

## 5 CONCLUSION

We assessed the genetic basis of SRA in a large panel of barley accessions and compared the differences between an Australian commercial breeding collection and a diverse collection of global gene‐bank germplasm. Using a haplotype‐based mapping approach, the study successfully identified two novel major genomic regions on chromosome 5H and confirmed a previously identified QTL on chromosome 3H, *RAQ1*, influencing SRA. The findings highlight the quantitative nature of the SRA trait and the likelihood of inadvertent selection through related polygenic traits within commercial breeding. To achieve significant improvement in barley in RSA and enhance adaptation, it is essential to harness predictive and precision breeding techniques that combine popular technologies such as genomic selection and gene editing.

## AUTHOR CONTRIBUTIONS

**Zachary Aldiss**: Conceptualization; data curation; formal analysis; investigation; methodology; writing—original draft; writing—review and editing. **Yasmine Lam**: Methodology; writing—review and editing. **Silvina Baraibar**: Resources; validation; writing—review and editing. **Sarah Van Der Meer**: Investigation; methodology; writing—review and editing. **Eric Dinglasan**: Investigation; validation; writing—review and editing. **Karen Massel**: Supervision; writing—review and editing. **Peter A. Crisp**: Supervision; writing—review and editing. **Ian Godwin**: Supervision; writing—review and editing. **Andrew Borrell**: Supervision; writing—review and editing. **David Moody**: Resources; writing—review and editing. **Lee Hickey**: Conceptualization; project administration; supervision; writing—review and editing. **Hannah Maria Robinson**: Conceptualization; project administration; supervision; validation; writing—review and editing.

## CONFLICT OF INTEREST STATEMENT

The authors declare no conflicts of interest.

## Supporting information




**Supplementary Figure 1**. Marker distribution and density of curated SNP markers across chromosomes 1H through to 7H of the barley genome.
**Supplementary Figure 2**. Pairwise marker LD‐matrices were calculated for each chromosome and LD‐decay was analysed showing an r2 of 0.2 with a sharp drop to 0.1 genome wide.
**Supplementary Figure 3**. Relationship between haploblock SNP density and haplotype effect variance. The x‐axis shows the number of SNPs per haplotype block, and the y‐axis represents the variance of haplotype effects within each block. No clear linear trend is observed, suggesting that the number of SNPs in a block does not directly predict the magnitude of haplotype effect variance.
**Supplementary Figure 4**. PCA of the AGG collection based on genome‐wide SNP variation. PC1 explains 29.5% and PC2 explains 13.9% of the total genetic variance. Individuals are coloured by passport information to reflect geographic origin: Africa (teal green), Americas (orange), Asia (purple), Europe (pink), Oceania (light green), United Kingdom (yellow), and individuals with no passport data (NA) are uncoloured.
**Supplemental Figure 5**. Physical positioning of EGT2 and marker identification. **(A)** Positioned within significant haploblock b001908, EGT2 (HORVU5Hr1G027890) was co‐located. (B) The closest SNP markers flanking EGT2 were identified at approximately 162.5 M (chr5H_00023624_161513719‐161674224_3547) and 159.5 M (chr5H_00023425_159395855‐159466194_9375) base pairs respectively. Both markers are approximately 1 M base pairs from the EGT2 locus. These markers were used to classify EGT2 haplogroups for subsequent analysis.
**Supplemental Figure 6**. EGT2 haplogroup frequency and association with SRA. **(A)** Haplogroups were created based on the marker information of the markers flanking EGT2 for each genotype. SNP combination CC‐CC was the largest frequency haplogroup followed by CC‐TT. (**B)** The highest frequency haplogroup was used as the reference for ANOVA to determine significant associations between haplogroups and SRA. CC‐TT was shown to have a significant association (p < 0.05), with an increased SRA of two degrees.
**Supplementary Figure 7**. Haploblock stacking for wide root phenotype. Each of the three panels displays the relationship between the number of beneficial haplotype blocks stacked (x‐axis) and the seminal root angle in degrees (y‐axis). The grey line represents the combined IGG and AGG populations, the orange line represents the IGG population alone, and the green line represents the AGG population alone. The plots illustrate the cumulative effect of stacking haplotypes associated with increased root angle, contributing to a wider root system architecture. The first panel considers the top 0.5% of haplotypes with the strongest positive effect on root angle, the second includes the top 10%, and the third includes all (100%) haplotypes.

## Data Availability

The data that support the findings of this study are available from InterGrain Pty Ltd, but restrictions apply to the availability of these data, which were used under licence for the current study, and so are not publicly available. Data are however available from the authors upon reasonable request and with permission of InterGrain Pty Ltd.
